# Essential Components of a Public Health Tuberculosis Prevention, Control, and Elimination Program: Recommendations of the Advisory Council for the Elimination of Tuberculosis and the National Tuberculosis Controllers Association

**DOI:** 10.15585/mmwr.rr6907a1

**Published:** 2020-07-31

**Authors:** Barbara Cole, Diana M. Nilsen, Lorna Will, Sue C. Etkind, Marcos Burgos, Terence Chorba

**Affiliations:** ^1^Riverside County Department of Public Health, Riverside, California; ^2^New York City Department of Health and Mental Hygiene, Long Island City, New York; ^3^National Tuberculosis Controllers Association, Smyrna, Georgia; ^4^Stop TB USA, Washington, DC; ^5^New Mexico Department of Health, Santa Fe, New Mexico; ^6^Division of Tuberculosis Elimination, National Center for HIV/AIDS, Viral Hepatitis, STD, and TB Prevention, CDC

## Abstract

This report provides an introduction and reference tool for tuberculosis (TB) controllers regarding the essential components of a public health program to prevent, control, and eliminate TB. The Advisory Council for the Elimination of Tuberculosis and the National Tuberculosis Controllers Association recommendations in this report update those previously published (*Advisory Council for the Elimination of Tuberculosis. Essential components of a tuberculosis prevention and control program. Recommendations of the Advisory Council for the Elimination of Tuberculosis. MMWR Recomm Rep 1995;44[No. RR-11]*). The report has been written collaboratively on the basis of experience and expert opinion on approaches to organizing programs engaged in diagnosis, treatment, prevention, and surveillance for TB at state and local levels.

This report reemphasizes the importance of well-established priority strategies for TB prevention and control: identification of and completion of treatment for persons with active TB disease; finding and screening persons who have had contact with TB patients; and screening, testing, and treatment of other selected persons and populations at high risk for latent TB infection (LTBI) and subsequent active TB disease.

Health departments are responsible for public safety and population health. To meet their responsibilities, TB control programs should institute or ensure completion of numerous responsibilities and activities described in this report: preparing and maintaining an overall plan and policy for TB control; maintaining a surveillance system; collecting and analyzing data; participating in program evaluation and research; prioritizing TB control efforts; ensuring access to recommended laboratory and radiology tests; identifying, managing, and treating contacts and other persons at high risk for *Mycobacterium tuberculosis* infection; managing persons who have TB disease or who are being evaluated for TB disease; providing TB training and education; and collaborating in the coordination of patient care and other TB control activities. Descriptions of CDC-funded resources, tests for evaluation of persons with TB or LTBI, and treatment regimens for LTBI are provided (Supplementary Appendices; https://stacks.cdc.gov/view/cdc/90289).

## Introduction

### Purpose

This report provides an introduction and reference tool for tuberculosis (TB) controllers regarding the essential components of a public health TB program. In addition, previously published guidelines are updated to provide a national standard for the assessment of individual TB control programs (*1*). This report also is a guide for public health TB programs, summarizing the essential components for a public health program to prevent, control, and eliminate TB (Box 1). Updates to clinical recommendations and other information that can change rapidly are provided online (Supplementary Appendices; https://stacks.cdc.gov/view/cdc/90289) but are not reflected in this published report.

BOX 1 **Summary of essential components for a tuberculosis prevention, control, and elimination program: recommendations of the Advisory Council for the Elimination of Tuberculosis and the National Tuberculosis Controllers Association**

**Role of public health departments**

Identify the unique role and responsibilities of the public health department in tuberculosis (TB) treatment and prevention.

**Overall planning and policy components**

Develop an overall TB control strategy, including written policies and procedures to provide guidance and oversight to facilities and practitioners involved in TB control.

**Surveillance and reporting of persons with suspected or confirmed TB disease**

Maintain a surveillance system for timely and accurate reporting of persons with suspected or confirmed TB disease.

**Data management, analysis, and use**

Conduct routine data collection and analysis of trends within the jurisdiction of the program, and apply the results to policy, planning, and prevention efforts.

**Program evaluation and quality improvement**

Evaluate programs, both internally and externally, to provide guidance for improvement.

**Laboratory and other testing**

Maintain access to laboratory and radiology tests recommended for TB disease, drug resistance, and TB infection.

**Identification, management, and treatment of persons with latent TB infection**

Identify, manage, and treat contacts and selected other persons infected with *Mycobacterium tuberculosis*.

**Identification, management, and treatment of persons with TB disease**

Manage persons with suspected or confirmed TB disease as soon as possible, begin a treatment regimen, and provide case management throughout treatment.

**Epidemiologic investigation**

Provide a thorough and timely investigation, whether investigating a source case or conducting a contact investigation.

**Training and education**

Ensure the provision of training and education to TB program staff, other health departments, clinicians, patients and families, community groups, and the general public.

**Partnerships and collaboration**

Work with stakeholders and populations at high risk to maximize efforts and minimize expenses. TB elimination cannot be accomplished by public health programs alone.

**Research**

Participate in local, national, and international research, as program capacity permits.

This report can be used in the following ways to optimize TB programs:

- To provide an introduction to TB control essentials for new TB controllers in state or local health jurisdictions
- To provide a frame of reference for policymakers and consultants for evaluating individual TB control programs
- To clarify and articulate the rationale for essential program components and activities to decision-makers, whether in health departments or in the legislative arena, who allocate financial and personnel resources to TB programs
- To educate colleagues or others regarding the structure and function of TB control programs

### Rationale for and Process of Updating the 1995 Guidelines

The recommendations in this report update those previously published regarding essential components of TB prevention and control programs (*1*). To update the report, a work group was formed comprising subject matter experts from the Advisory Council for the Elimination of Tuberculosis (ACET), the National Tuberculosis Controllers Association (NTCA), and CDC. The report has been written collaboratively on the basis of experience and expert opinion on approaches that have succeeded in organizing programs engaged in diagnosis, treatment, prevention, and surveillance for TB at state and local levels. Each member of the work group was assigned sections to write. Regularly scheduled meetings were held to revise or review the report. The finalized report was approved by ACET, constituting authorities knowledgeable in the fields of public health, epidemiology, immunology, infectious diseases, pulmonary disease, pediatrics, tuberculosis, microbiology, and preventive health care delivery, and representation of the stakeholders, namely state, territorial, and local health departments and other federal agencies and organizations with interests in TB prevention and control.

The recommendations have been updated as a result of changes in epidemiology of TB in the United States, where incidence rates decreased or plateaued during 1993–2019 (*2*), and changes in technologies that assist in diagnosis, treatment, prevention, and surveillance of disease. In certain jurisdictions, this progress has led to changes in the organization of and to decreased funding for TB programs, which might impede additional progress toward elimination of TB as a public health problem in the United States.

In 1993, the World Health Organization (WHO) declared TB to be a global emergency, as connections between the then-emerging human immunodeficiency virus (HIV) epidemic and TB were being recognized, with persons with HIV infection having marked increases in risk for developing TB relative to those without HIV infection (*3*). The resulting increases in TB incidence and prevalence had global implications. By 2014, when annual TB deaths worldwide reached 1.5 million, WHO formulated an action framework, thus providing guidance toward ending the ongoing global TB epidemic, progress of low-incidence countries to preelimination by 2035, and subsequent progress in these same countries toward achieving elimination of TB as a public health problem by 2050 (*4*).

CDC has a national strategic TB plan (*5*), and in 2015, the U.S. government released an action plan specifically addressing drug-resistant TB (*6*). This report presents the essential TB program elements needed in U.S. public health jurisdictions to maintain progress toward national and international objectives. This report also provides guidance for the increasing number of jurisdictions that, in coming years as TB epidemiology in the United States continues to evolve, will be able to transition from traditional TB control efforts to additional activities that move toward TB elimination.

In 1988, the Institute of Medicine report *The Future of Public Health* (*7*) proposed a conceptual framework consisting of three core functions of public health agencies at all levels of government: assessment, policy development, and assurance. Subsequently, the Core Public Health Functions Steering Committee proposed a list of the 10 essential public health services that could be mapped to those three core functions (*8*). This report can be viewed within the framework of core public health functions and essential services. These lists of core functions and essential services were not formulated in a TB-specific context but rather were meant to be so general in conceptual scope that their framework could be broadly applicable to any public health problem. TB controllers might find that this core function framework provides a useful conceptual structure through which to provide initial briefings and education about the fundamentals of TB disease, TB control, and TB elimination to potential partners and decision-makers. The terminology more commonly used by TB control professionals can be usefully mapped to the core functions. Specific details regarding the core public health functions and essential services are provided (Supplementary Appendix B; https://stacks.cdc.gov/view/cdc/90289).

### Brief History of TB in the United States

TB incidence in the United States has decreased considerably from the early 1950s through 2020. Although 84,304 cases were reported in the United States in 1953, only 8,920 were reported in 2019 (*2*). In addition, although the U.S. population doubled during those years, TB case rates decreased more than eighteenfold, from a rate of 52.6 cases per 100,000 population in 1953 to 2.7 in 2019. With global TB incidence at approximately 130 cases per 100,000 population, the United States and multiple other countries are considered to be countries with low TB incidence. However, the generally favorable long-term declining trend was interrupted in the mid-1980s and early 1990s, initially by a 5-year plateau in incidence (1984–1988), followed by a 4-year period (1989–1992) during which TB incidence increased by 14%. This resurgence of TB in the United States was attributed to factors that included all of the following (*9*,*10*):

- Immunosuppression: the HIV epidemic that began in the 1980s
- Immigration: the increasing occurrence of TB among non–U.S.-born persons from countries with a high prevalence of TB
- Inadequate infection control: widespread transmission of *Mycobacterium tuberculosis*, the causative bacterium of TB, in congregate settings (e.g., health care facilities, correctional facilities, drug treatment centers, and homeless shelters)
- Reductions in funding: reductions in funding for TB programs over several decades as a result of the widespread perception that trends in TB incidence during the 1950s, 1960s, and 1970s indicated that TB incidence was substantially reduced

Resurgence of TB eventually led to increased federal funding, which along with the widespread adoption of the essential TB program components, contributed to improvements in TB prevention and control efforts. These improvements have led to decreasing TB incidence rates. Thus, when the ACET recommendations were published in 1995 (*1*), the principal challenge for state and local TB programs was to regain control and reverse the resurgence of TB. However, the challenge now faced by certain health jurisdictions is continuing to manage and prevent TB successfully during an era in which 1) decreasing incidence might lead to decreasing visibility and resources and 2) new approaches to diagnosis, surveillance, treatment, and prevention are needed because of the changing epidemiology of TB in the United States.

In 2000, the Institute of Medicine issued the report *Ending Neglect: The Elimination of Tuberculosis in the United States* (*11*), which outlined a strategy that included the following six goals:

- Maintain disease control
- Accelerate the decrease in incidence
- Develop new prevention, control, and treatment tools
- Address global TB incidence
- Mobilize and sustain public health support
- Track elimination progress

The strategies are still relevant and should be implemented if the United States is to meet its TB elimination goal.

## Strategies for TB Prevention and Control

This report reemphasizes the importance of three priority strategies for TB prevention and control that have been well established for decades:

1. Identification of and completion of treatment for persons with active TB disease to render their condition noninfectious
2. Finding and screening persons who have had contact with TB patients to determine
  - whether they have active TB disease themselves;
  - whether they have been infected with *M. tuberculosis;* or
  - for children and other persons at high risk, whether they require window prophylaxis (preventive treatment of presumed TB infection during the time that it would normally take for a tuberculin skin test [TST] or interferon gamma-release assay [IGRA] to become positive after exposure) and whether to administer treatment
3. Screening, testing, and treatment of other selected persons and populations at high risk for latent TB infection (LTBI) and subsequent active TB disease to detect persons who can most benefit from treatment for LTBI, which is essential for TB elimination because of new immunosuppressive drugs and therapies used for different illnesses, immigration from areas where TB is endemic, and diminished knowledge and reduced recognition of TB by clinicians as a result of decreased incidence

Although the three priority strategies focus on individual patients, they serve broader epidemiologic functions because they reduce current and future transmission of *M. tuberculosis* in the community.

### Essential Components

Health departments are responsible for public safety and for protecting population health (Box 2). Through cooperative agreements with CDC for TB prevention and control activities, state, local, and tribal health departments have primary responsibility for ensuring that the three priority strategies are implemented in their respective jurisdictions (*12*). To meet these responsibilities, TB control programs should be able to institute the following essential components of responsibilities and activities (Box 1) or ensure that they are completed:

BOX 2 **Legal framework for public health departments in tuberculosis prevention and control**

Health departments are charged with public safety and with advancing and protecting population health. They are authorized and compelled by legal statutes to perform specific duties to protect the public’s health. International and national standards define basic principles that guide health departments and represent a minimum practice for ensuring success in protecting populations from tuberculosis (TB). Although multiple entities perform activities that reduce the spread of TB (e.g., evaluating and treating TB infection and disease), actions that are taken by health departments are time-sensitive, are supported by a jurisdiction’s legal framework, and focus beyond the individual level to safeguard the community’s health. The unique scope of services and responsibilities of TB control is bound by a legal framework assigned to federal, state, local, and tribal health departments. This legal framework includes investigating persons with TB and persons exposed to TB, responding to TB outbreaks, enforcing TB reporting, and issuing orders of isolation, evaluation, and treatment.

**Source:** Hodge Jr JG, Anderson E, Nelson G, Larson M. Tuberculosis control laws and policies: a handbook for public health and legal practitioners. Atlanta, GA: US Department of Health and Human Services, CDC; 2009. https://www.cdc.gov/tb/programs/TBlawPolicyHandbook.pdf

- Preparing and maintaining an overall plan and policy for TB control
- Maintaining a surveillance system for timely and accurate reporting of persons with suspected or confirmed TB disease
- Collecting and analyzing data for assembling local, state, and national statistics and reporting to stakeholders
- Participating in program evaluation and research to advance local, national, and international knowledge of the best ways for identifying, treating, and preventing TB
- Prioritizing TB control efforts, especially in settings in which resources are unavailable for all possible proposed activities
- Ensuring access to laboratory and radiology tests recommended for TB disease, drug resistance, and LTBI
- Identifying, managing, and treating contacts and other persons at high risk for infection with *M. tuberculosis* to prevent TB disease and move toward elimination
- Managing persons who have TB disease or who are being evaluated because they might have TB disease
- Providing training and education for TB program staff, other health departments, clinicians, patients and their families, community groups, and the general public
- Collaborating with medical, community, and academic partners; with TB colleagues in adjacent and other jurisdictions; and with staff from selected other public agencies (e.g., corrections, social services, and immigration and naturalization) to coordinate patient care and other TB control activities

A summary of the components of TB-related responsibilities and activities of health departments is provided (Box 3), and the responsibilities of the TB controller or TB program manager are described (Supplementary Appendix A; https://stacks.cdc.gov/view/cdc/90289). By implementing the activities described in this report, TB programs can increase their effectiveness in controlling the disease and increase their jurisdiction’s ability to move beyond control and toward elimination.

BOX 3 **Components of responsibilities and activities of public health departments in tuberculosis prevention and control: recommendations of the Advisory Council for the Elimination of Tuberculosis and the National Tuberculosis Controllers Association**

- Assist in development, implementation, and evaluation of tuberculosis (TB) screening, testing, and treatment programs for both latent TB infection (LTBI) and TB disease.
- Establish priorities for prevention and control activities.
- Protect the public’s health by isolating and treating persons with infectious TB disease.
- Involve other health care providers in screening, testing, treatment, and preventive treatment activities.
- Identify and establish working relationships with persons, facilities, and agencies providing health care services to populations at high risk. Assist these service providers in developing, implementing, and evaluating programs tailored for the community’s needs.
- Provide support for staff training.
- Identify medical consultants with expertise in TB patient management.
- Arrange referrals and monitoring for persons who have TB disease or adverse reactions while on therapy for LTBI or TB disease.
- Review and evaluate TB control activities. Periodic assessments are needed for examining their effectiveness.
- Potentially act as primary provider for treatment and evaluation of patients with TB disease or LTBI, depending on the jurisdiction.
- Ensure that the responsibilities of both the TB controller and the TB program manager are performed (Supplementary Appendix A; https://stacks.cdc.gov/view/cdc/90289).

### Additional Strategies for Progressing Toward TB Elimination

Although many of the general program components previously described have remained unchanged, the context in which these components are applied has changed. Thus, as some jurisdictions note decreasing numbers of cases and TB case rates, they should increasingly focus on additional strategies that can accelerate progress toward TB elimination. Such strategies might include the following:

- Making LTBI, in addition to suspected and active TB disease, a reportable condition
- Providing directly observed therapy (DOT) not only to TB patients but also to selected persons with LTBI
- Enlisting partners (e.g., private medical practitioners, student health services, employers of non–U.S.-born persons, hospitalist physicians, and staff from other non-TB health department programs) to broaden screening for LTBI and to record results in a standardized manner
- Analyzing case and infection data at least annually to provide guidance and allow targeting of subsequent testing and treatment efforts
- Maintaining awareness of both the incidence of TB disease and the prevalence of LTBI for 1) the public through news releases and posting of information online and 2) health professionals through published case reports, case series, epidemiologic analyses of local TB data, and presentations to state, local, and tribal medical societies and hospital staff
- Maintaining communication with partners and collaborators in other local, neighboring, state, tribal, and federal agencies to build collaborative TB initiatives

## Overall Planning and Policy Components

### Overall TB Control Strategy and Written Policies and Procedures

As part of the overall TB control strategy, TB programs should consider the changing health care environment in which TB care is provided. Policies and procedures should align with the core public health functions and essential services (Supplementary Appendix B; https://stacks.cdc.gov/view/cdc/90289). Evolving technologies (e.g., electronic health records, telemedicine, and electronic platforms for remotely viewing patients taking their medications) provide alternative methods for facilitating care. Cross-cutting concerns (e.g., ensuring an uninterrupted supply of TB medications, adequate funding, and modification or development of new laws to support TB control) should be addressed at the state and national levels. Also important is detecting and treating TB disease while addressing the reservoir of persons with LTBI.

TB control programs should develop an overall TB control strategy in collaboration with local partners (e.g., health care providers, professional societies, and voluntary organizations) and state, local, and tribal advisory committees, if available. To determine specific needs, national, state, local, and tribal TB morbidity data and standard indicators of program performance (e.g., the CDC National Performance Objectives) should be used (*13*).

The TB control strategy should outline program priorities and objectives that consider epidemiologic data and the roles of the partnering agencies, organizations, and providers. TB control programs should also have written policies and procedures that clearly define the standard of practice for TB treatment and prevention. These priorities, objectives, and plans should be reviewed periodically and revised as needed.

### Advising Health Department Clinics, Local Institutions, and Health Care Providers

TB control programs should provide consultation and oversight for TB-related activities throughout their jurisdictions, regardless of who might be providing services. This consultation and oversight will help ensure these services reflect standards of care and public health practice. Programs should maintain expertise in TB management and have access to medical consultation when needed. Pediatric-specific expertise should be available to all TB programs and community providers. Information might be available either from health department staff or from designated experts who have agreed to act as consultants for the health department. Consultants should be available to advise health care providers, agencies, or institutions about routine challenges in patient management (e.g., treatment adherence, interpretation of radiologic tests, and common adverse drug reactions) and complex clinical situations.

Programs should ensure that staff or external consultants are also available to provide guidance regarding

- use and interpretation of IGRAs;
- genotyping;
- mycobacteriology laboratory methods, including new molecular diagnostics;
- molecular drug-resistance testing;
- contact or outbreak investigations;
- new technologies and new medications as they become available and how to use them; and
- other public health concerns within TB control in both community and institutional settings.

### Collaboration with CDC

In collaboration with state, local, and tribal health departments, CDC’s Division of Tuberculosis Elimination (DTBE) serves as the national TB program, providing support and guidance to all TB programs in the United States. CDC’s Division of Global Migration and Quarantine (DGMQ) also supports TB programs by preventing the introduction, transmission, and spread of infectious diseases into the United States. DTBE’s TB program consultants are responsible for coordinating and assisting TB programs in all 50 states, eight current or former territories, and nine large cities. In addition, CDC performs the following activities:

- Administers and promotes a national program for preventing, controlling, and eliminating TB
- Provides leadership and formulates national policies and guidelines
- Conducts behavioral, health systems, and clinical research
- Supports a nationwide framework for TB surveillance and evaluation of national TB prevention and control program performance
- Provides administrative support for the federal TB task force and supports and collaborates with NTCA to promote effective national communications and coordinated feedback regarding urgent policy and program performance concerns
- Provides technical supervision and training to federal assignees working in the state, local, and tribal TB control programs
- Develops training and educational materials and provides technical assistance for communications and training needs
- Funds TB Centers of Excellence for Training, Education, and Medical Consultation in strategic geographic locations
- Participates in developing policies and guidelines for TB prevention and control among populations at high risk (e.g., persons with HIV infection)
- Provides programmatic consultation, technical assistance, and outbreak response assistance to state, local, and tribal health departments and provides technical assistance to TB programs in other countries by collaborating with international partners

CDC guidelines, publications, educational materials, and contact information are available from CDC’s TB website (*14*), and information on CDC-funded resources is available (Supplementary Appendix C; https://stacks.cdc.gov/view/cdc/90289).

### Infection Control

TB control programs should serve the medical community as sources of information and consultation regarding infection control practices for TB (*15*). During interactions with the medical community, TB control programs should emphasize the need for maintaining a high level of suspicion for TB when evaluating patients who have TB symptoms and also the importance of early diagnosis, isolation, and prompt therapy initiation. The programs should give expert advice or provide referrals to experts for information about infection control measures for different settings (e.g., hospitals, clinics, nursing homes, correctional facilities, homeless shelters, and drug treatment centers). Guidance for how to use new diagnostic tests for release from airborne isolation in hospitals is now available (*16*); use of these techniques is specific to each case and should be in accordance with local TB control requirements.

TB control programs should educate facility staff providing care for TB patients about the need for routine periodic evaluation of infection control practices and might also assist in the evaluation process. Assistance can include providing updated recommendations and regulations to the facility, providing names of experts in infection control, or providing access to personnel involved in programmatic evaluations.

TB increases in certain geographic areas are related to the high risk for TB among immunosuppressed persons who have HIV infection. Transmission of *M. tuberculosis* to HIV-infected persons is of particular concern because, if coinfected with *M. tuberculosis*, these persons are at higher risk for the rapid development of TB disease (*17*). Program staff should be particularly alert to the need for preventing transmission of *M. tuberculosis* in settings where HIV-infected persons work or receive care. Administrative controls should be established on the basis of CDC, federal, and state guidelines (*18*). This might include surveillance for TB exposure among employees or fit testing of respirators for certain employees.

## Laws, Regulations, and Policies to Support TB Control Activities

TB control programs should periodically review applicable laws, regulations, and policies to ensure their consistency with recommended medical and public health practices. Jurisdictions should recommend laws and create regulations and policies that provide a legal basis for TB control activities. Such laws and regulations should include

- ensuring prompt, mandatory reporting of each confirmed and suspected case of TB disease;
- protecting the health of the public by isolating and treating persons who have infectious TB;
- rapidly detaining persons who have begun TB treatment and achieved noninfectious status but who are unwilling or unable to complete treatment and are at risk for reverting to or developing an infectious status; and
- observing laws and regulations protecting patient confidentiality.

Policies should address

- ensuring examination of persons at high risk for TB disease and the prescription and monitoring of treatment for these persons;
- ensuring rapid laboratory examination of specimens and reporting of results, including drug-susceptibility test (DST) results and negative culture results, to the health department and the requesting clinician;
- ensuring that communication between providers and the health department is established for all hospitalized patients;
- ensuring that patients who have TB disease receive treatment until they are cured, possibly including mandated DOT;
- treating patients regardless of their ability to pay; and
- encouraging health care facilities and congregate settings to apply recommended measures for infection control.

TB programs can educate policymakers or provide technical assistance regarding laws, regulations, and policies that mandate activities that are no longer recommended, either by implementing authoritative guidelines or by carefully reviewing epidemiologic data and research.

### Adequate and Qualified Staff for Conducting TB-Related Activities

TB control programs should have adequate and well-trained staff for performing the TB-related activities outlined in this report. The number and type of staff needed by programs will vary depending on local TB morbidity, the structural organization of the public health activity, and specific community needs. Each jurisdiction determines its own staffing patterns and needs. Staff are necessary for providing medical consultation and for such activities as program planning, personnel and financial management, record-keeping, collection and epidemiologic analysis of surveillance and other program data, staff and patient education and training, social services, and coordination of TB-related activities with other health department activities in the jurisdiction.

All TB control programs should have a designated program manager. Depending on how a program is organized, the program manager might also be the state’s designated TB controller. Programs might also have nurses, physicians, medical consultants, public health advisors, epidemiologists, outreach workers, and other staff as needed to provide essential TB program services.

Policies and procedures that clearly define the roles and responsibilities of each member of the TB program staff will facilitate training and evaluation of each member. In certain settings, assistance for studies or special projects might be available from students in such fields as epidemiology, health promotion, public health, nursing, or other related fields or from physicians or medical students, especially those interested in infectious disease or pulmonary medicine, who might spend some of their elective time working with a TB program.

Creating such opportunities for students can benefit TB programs. Policies and procedures regarding the work responsibilities, access to confidential data, and publication rights should be in place before students work with a TB program. TB programs should clearly define the participation of students with direct patient care activities (e.g., TB clinic, home visits, or contact investigations). Nursing students should function within their scope of practice. Masters of public health interns can assist with data analysis and program evaluation.

A TB clinic might be part of the TB control program, with staff who include different combinations of nurses, physicians, physician assistants, and other workers. Clinics might have nurse managers responsible for providing the majority of the education, treatment, clinical monitoring, prevention services, and supervisory needs of the clinic. A physician who is qualified and trained in TB diagnosis, management, and clinical monitoring should be available on staff or employed on a contract basis. Clinic staff should have cross-cultural skills and receive ongoing training appropriate to the community’s cultural and language needs. Interpreter services are legally required when providing health care; programs should work with all health care providers to ensure that these services are available. Clear policies and procedures for each role within the clinic will delineate responsibilities and help define training and evaluation.

In settings where medical care is provided by private-sector practitioners, the TB program should coordinate services between those health care providers and the public health staff providing DOT and daily monitoring of persons with TB disease. Policies and procedures should define the collaboration process so that all parties know what is expected of them.

### Funding for Conducting TB Control Activities

TB control in the United States is the legal responsibility of state and local governments (*19*); financing of TB control programs in the United States relies on a mixture of state and local funding, with supplementary funding provided by the federal government. All 50 states, nine large cities, five territories, and three affiliated sovereign Pacific Island nations (Palau, the Federated States of Micronesia, and the Republic of the Marshall Islands) receive federal supplementary funding directly from CDC. Funding formulas are applied by CDC to allocate supplementary funding to these funded jurisdictions through cooperative agreements. These formulas are both needs based (based on average case numbers in the jurisdictions) and performance based (e.g., numbers of patients completing treatment within 1 year and mean volume of tests performed), focusing on prevention and control activities and on the laboratory systems. A prevention and control funding formula is based on TB surveillance data using variables with universal and consistent reporting by all of the 67 funded jurisdictions in the National Tuberculosis Surveillance System (NTSS). The federal allocation of supplementary TB funding is based on an iteration of the funding formula that has both incidence (80%) and performance (20%) components. A workload-based laboratory formula determines the federal allocation of supplementary support for TB mycobacteriology work in 58 public health laboratories.

TB program staff should consult CDC program staff regarding specific funding requirements. TB control programs should seek funding for TB control activities from federal, state, local, tribal, and private sources. They also should work with local organizations (e.g., state and local medical societies, lung associations, community groups, and TB coalition members) to educate policymakers about TB epidemiology, local program priorities, needs, and objectives to ensure TB control. Policies and procedures regarding acceptance and use of funds should be consistent with federal, local, and state requirements.

### Collaboration with Entities Outside the Health Department

#### Communities with High TB Prevalence

Optimal TB prevention and control activities require a multidisciplinary approach (*18*). Thus, TB control programs in communities with a high prevalence of TB should form networks and coalitions with local groups (e.g., cultural and ethnic organizations, community clinics, places of worship, professional societies, lung associations, and medical and nursing schools). Collaboration with these groups helps the TB control program ensure that community leaders, health care providers, and policymakers are knowledgeable about TB; educate the public about TB; and provide guidance and assistance for local screening and prevention services.

Coalitions with community groups help TB control programs reach groups at high risk more effectively and provide culturally appropriate services. TB control programs should educate and advise community groups to ensure the quality and appropriateness of TB-related activities in accordance with the community’s needs and national performance standards. Policies regarding definition of roles, access to confidential information, and responsibilities for different parts of a collaborative program will ensure that all participants know how each collaboration works. Stop TB USA (*20*) exemplifies a national coalition involved in educating health care providers and the public about TB.

#### Correctional and Other Congregate Settings

Although occurrence of TB in correctional facilities and other congregate settings varies from state to state, any delay in detecting a person with infectious TB continues to represent a public health threat. TB control is an essential element in health care in correctional facilities. All correctional facilities, including those in which few TB cases are expected to occur, should designate a person or group of persons experienced in infection control, occupational health, and engineering who are responsible for the facility’s TB infection control program.

TB infection control officials should be familiar with TB control and prevention practices that include screening, containment, and assessment to ensure prompt identification of persons who might have infectious TB. More detailed descriptions of these practices is integrated throughout this report.

Protocols should exist for diagnosis, treatment, and follow-up within congregate settings. TB programs should ensure that all clinicians who treat inmates or employees with LTBI or active TB disease are familiar with CDC-recommended treatment protocols. Public health departments should assist correctional and other congregate facilities as needed with developing and updating policies and procedures for TB control. The health department should provide access to expert TB medical consultation, adequate laboratory services, and consultation or assistance with epidemiologic investigations for major TB exposures in congregate settings.

Homeless shelters are another congregate setting with potential for TB transmission if persons with infectious TB are not identified in a timely manner. Screening, containment, and assessment activities can present special challenges caused by the mobile nature of the population. Health departments should ensure that epidemiologic investigations are conducted, whether by the health department or the shelter facility.

### Research, Data Analysis, and Interpretation

TB programs should generate data for use in program evaluation, planning, and implementation of data-driven initiatives. Each program should have policies and procedures for confidential and nonconfidential data access and use of analyzed data. TB programs can benefit by participating in research regarding TB, whether by working with a local student to support a master’s thesis or participating in national and international studies. Before participating, policies should be in place regarding participation in research, designation of an institutional review board, and clarification of responsibilities for supervision of research students.

## Surveillance and Reporting of Persons with Suspected or Confirmed TB Disease

### Surveillance

TB surveillance data are essential for designing public health interventions that accelerate progress toward TB elimination in the United States. Identification of affected populations, geographic distribution of cases, comorbidities, and LTBI trends provide the foundation for culturally appropriate interventions. Monitoring DST results at the local, state, tribal, and national levels is a vital surveillance activity. In addition, DSTs are needed for ensuring correct treatment regimens. Collection of TB data at the local, state, tribal, and national levels is based on morbidity and mortality reports. Although reporting laws vary from state to state, TB is a nationally reportable condition; for each identified case of active TB disease, the report of a verified case of TB (RVCT) should be submitted to CDC (*21*).

#### Drug-Resistance Surveillance

TB control programs should ensure that surveillance on drug resistance is performed on all initial isolates of *M. tuberculosis* and that the results are reported promptly to the primary care provider and the local health department and are submitted electronically to CDC through the RVCT form. Drug-resistance surveillance includes genetic mutation analysis as well as DST. TB control programs should monitor local drug-resistance patterns and rates to assess the effectiveness of local TB control efforts and to determine the appropriateness of the CDC-recommended initial TB treatment regimen for any patient.

#### Reporting Confirmed Cases and Suspected Cases 

TB control programs should ensure and facilitate reporting of TB cases and suspected TB cases from hospitals, health care providers, laboratories, and other sources, depending on state or local laws. TB control programs should monitor the completeness of reporting and the duration of time between diagnosis and reporting. Programs should communicate regularly with infection control practitioners in hospitals, clinics, or congregate settings where TB is frequently diagnosed or that tend to serve populations at high risk.

TB cases and suspected TB cases should be reported and entered promptly into the electronic case database and transmitted to CDC as required. Prompt TB case or suspected TB case reporting is necessary for

- evaluation, treatment, and case management;
- effective contact investigations;
- treatment completion for patients and their infected contacts;
- identification and prevention of ongoing transmission;
- program planning and evaluation at local, state, tribal, and national levels; and
- monitoring of epidemiologic trends, including drug resistance and identification of populations at risk.

Data from case reports should be reported promptly to NTSS and submitted electronically to CDC. These data are used for monitoring national TB trends, prioritizing needs, and creating the annual CDC TB surveillance report (*22*).

#### Case Finding

Passive and active surveillance are principal strategies for identifying TB cases. Passive surveillance relies on health care providers or laboratories reporting persons with clinical, radiologic, or bacteriologic findings indicative of TB. TB control programs should ensure health care providers and laboratories are familiar with and follow disease reporting laws.

To identify unreported cases, TB programs should conduct active surveillance focusing on health care providers serving populations at high risk, laboratories, and pharmacies. Unreported cases might also be discovered through routine checks of death records and Medicare and Medicaid billing. TB control programs should understand their local epidemiology to identify populations at high risk and engage in appropriate case finding and prevention measures. Genotyping of specimens that are culture positive for *M. tuberculosis* can identify clusters of patients where transmission might have occurred and can be used to monitor for TB outbreaks. TB testing of populations at low risk should be discouraged because it can yield false-positive results and strain limited public health resources.

### TB Case Registry

To fulfill community public health responsibilities, TB control programs should maintain a computerized record system (case registry) with updated information on all clinically active and suspected TB cases in the community. To ensure follow-up with all TB patients and persons with suspected TB, registry information (e.g., smear, culture, and DST results; clinical status; chest radiograph results; and doses of medications being administered) should be regularly obtained and updated. A specified health department staff member should review detailed registry information routinely to 1) identify patients with confirmed TB disease or LTBI and who might be having difficulties adhering or responding to standard therapy (e.g., patients who have persistently culture-positive sputa or who are taking medications to which their TB organisms are resistant, and 2) ensure follow-up (e.g., initiating field follow-up visits or arranging medical consultation with providers).

TB control programs should also maintain records regarding screening, examination, and treatment status of contacts of patients with infectious TB. Program staff should periodically review screening activities to assess their effectiveness in identifying persons with LTBI and ensure that these persons are completing courses of treatment for LTBI when recommended. If reviews demonstrate that few or no new cases are being identified by particular screening activities, TB programs should further evaluate or discontinue these activities as indicated.

### Transfer of Information Among Jurisdictions

Persons with TB disease or LTBI sometimes move among health jurisdictions. The TB program should facilitate information transfer among jurisdictions so that patients are not lost to follow-up and that treatment is continuous and completed. A sample interjurisdictional report form is available on the NTCA website (*23*).

### Reporting of LTBI Cases

Although in certain jurisdictions all LTBI cases are reportable, information should be reported promptly regarding contacts of patients with infectious TB in any jurisdiction. A surveillance case definition for LTBI approved by the Council of State and Territorial Epidemiologists can be used (*24*).

Close contacts of persons with infectious TB, and other contacts at high risk for infection, should be tested for TB infection as soon as they can be identified; if contacts within this group are infected, TB programs should evaluate the need to expand the contact investigation so that persons potentially infected are able to be tested and, if found infected, medically evaluated and offered treatment if indicated. Persons recently infected are at the highest risk for progression to TB disease; therefore, complete and rapid reporting of their status and initiation of treatment are necessary for preventing the spread of TB disease.

TB programs should identify other groups at high risk and collect their testing results to establish a baseline for an infection rate. Groups at high risk who are reported to state or local health departments on arrival in the United States include immigrants and refugees with a class B notification status. Class A and B notification categories can be ascribed to visa applicants by panel physicians in the country of origin (Box 4) (*25*). TB programs should strive to screen, test, evaluate, and offer treatment to those persons, if indicated.

BOX 4 **Tuberculosis classifications and travel clearance**

**Class A tuberculosis (TB) disease**

This class includes all applicants who have TB disease. This also includes applicants with extrapulmonary TB who have a chest radiograph indicative of pulmonary TB disease, regardless of sputum smear and culture results.

**Class B0 TB, pulmonary**

This class includes applicants with diagnosed TB by the panel physician or who were seen by the panel physician while on TB treatment and who have successfully completed directly observed therapy (DOT), as defined by CDC’s Division of Global Migration and Quarantine, under the supervision of a panel physician before immigration.

**Class B1 TB, pulmonary**

This class includes applicants who have signs or symptoms, physical examinations, or chest radiograph findings indicative of TB disease or who have known human immunodeficiency virus (HIV) infection but have negative acid-fast bacillus sputum smears and cultures and do not have diagnosed TB disease. This classification also includes applicants who have TB disease diagnosed by the panel physician, have refused DOT treatment, or have returned after treatment and completion of a 1-year waiting period.

**Class B1 TB, extrapulmonary**

This class includes applicants with diagnosed extrapulmonary TB with a normal chest radiograph and negative sputum smears and cultures.

**Class B2 TB, LTBI evaluation**

This class includes applicants who have a positive interferon gamma-release assay (IGRA) or tuberculin skin test (TST) but otherwise have a negative evaluation for TB. Contacts with a positive IGRA or a TST of ≥5 mm induration must receive this classification in addition to a class B3 TB, contact evaluation, classification (if they are not already class B0 TB, pulmonary; class B1 TB, pulmonary; class B1 TB, extrapulmonary; or class A TB [with TB disease]).

**Class B3 TB, contact evaluation**

This class includes applicants who are a recent contact of a known TB disease patient, regardless of IGRA or TST results. If the IGRA or TST is positive and no evidence of TB disease exists, two classifications apply: class B2 TB and class B3 TB; if negative, only class B3 TB applies.

**Source:** CDC. Tuberculosis technical instructions for panel physicians. Atlanta, GA: US Department of Health and Human Services, CDC. https://www.cdc.gov/immigrantrefugeehealth/exams/ti/panel/tuberculosis-panel-technical-instructions.html

The U.S. Preventive Services Task Force (USPSTF) issued a recommendation for screening for LTBI among populations at increased risk for TB (*26*). When primary care practitioners implement this recommendation, they might increase the number of persons treated for LTBI and potentially decrease new cases of TB disease. A method for clinical health care systems to track and report their screening, testing, and treatment would supplement the tracking and reporting system used by public health departments.

## Data Management, Analysis, and Use

Data, management, analysis, and use for reporting, quality improvement, identification of epidemiologic links, and reporting to stakeholders are vital public health functions. Data should be validated before analysis and use, and quality-control measures should be established to ensure the accuracy of both the data and data entry.

The ability to use all relevant data available about TB cases is necessary for investigating thoroughly and then preventing TB disease. Genotyping, whole-genome sequencing, and drug-susceptibility patterns can help providers identify links between persons with TB disease that are not evident through questioning the patient to identify contacts. These links might provide additional information about others at risk for TB disease.

### Protection of Patient Confidentiality

TB control programs should develop policies for ensuring data security and confidentiality of TB-related records. TB programs should enact strategies for protecting all TB reports, records, and files containing patient names or other identifying information. Local policies regarding the security and confidentiality of such information, including HIV test results, should adhere to all applicable laws. TB programs should collaborate with HIV programs and other relevant disease control programs in developing and implementing confidentiality policies.

### Data Transmission and Storage

TB programs should store TB data securely in an electronic system that allows data to be transmitted to care providers, other health departments, and consultants, as needed. These data should include not only case notes and medication records but also digital radiographic films (if feasible), interpretations, laboratory results, and information about drug resistance.

## Program Evaluation and Quality Improvement

### Importance of Ongoing Program Evaluation

Ongoing and systematic program evaluation activities are an integral component of public health program success. As TB program resources have decreased during the past decade, the need for ongoing program evaluation has acquired additional importance because finite resources should be applied to the most effective program activities. Measuring the impact and effectiveness of individual activities within TB programs serves to facilitate continuous improvement and to examine the need for new or ongoing program activities. When developing a TB program or activity, staff should consider identifying a baseline before implementing the activity, particularly when that program is expected to be ongoing. Reports and data are available from CDC’s TB website (*27*) and are detailed in this report (Supplementary Appendix C; https://stacks.cdc.gov/view/cdc/90289).

### Internal Use of TB Program Evaluation

In addition to analysis of surveillance data for monitoring morbidity trends, programs can determine the demographic characteristics of their patient population, monitor drug-resistance rates, and determine treatment outcomes. Additional analyses regarding effectiveness and outcomes of contact investigations and targeted testing for LTBI and treatment programs should be performed. These analyses can be used to assess program performance and progress toward achieving locally and nationally established program objectives.

### National Tuberculosis Indicators Project

Since 2005, CDC has included program evaluation as a core requirement of its cooperative agreements with TB programs. With the understanding that TB programs face resource limitations and other constraints, CDC developed the National Tuberculosis Indicators Project (NTIP) (*28*), a monitoring system for tracking TB program progress toward meeting the U.S. national TB program objectives.

NTIP facilitates use of existing data to help programs prioritize activities and focus program evaluation efforts. NTIP provides a standardized method for calculating indicators and tracking program progress across sites and temporally, thus enabling the ability of CDC and TB programs to assess the impact of TB control efforts. NTIP uses data that are being reported to CDC through the RVCT form (*21*), the Aggregate Reports for Tuberculosis Program Evaluation (*29*) for patients’ contacts, and the Electronic Disease Notification System (*30*) for follow-up evaluation of immigrants and refugees with class A or B conditions (Box 4). TB programs are encouraged to share NTIP and other program evaluation reports with relevant public, private, and community groups. TB programs should engage transparently with these groups in developing evidence-based efficient and effective strategies for sustaining TB prevention and care efforts.

### Cohort Review

The cohort review process is a systematic review of management of TB patients and their contacts. Cohort review identifies areas of success and those that need improvements in programmatic and clinical operations. A cohort is a group of TB patients identified during a specific time, often 3 months, who have completed or are nearing the end of treatment. Details regarding patients’ clinical status, case management, respective contact investigation, and outcomes are reviewed by staff, case managers, and clinicians to ensure accountability, educate staff about products and goals, and improve case management and prevention. Programs should review TB cases among children and other sentinel events as separate cohorts. Details of the cohort review process are available from CDC’s TB website (*31*).

### Registry Reviews

Programs also should conduct periodic reviews of case registry and other selected data records systems (e.g., laboratory reports, pharmacy reports, acquired immunodeficiency syndrome registries, and death certificates) to validate the surveillance system and to detect any failure to report cases. Such periodic reviews assist in identifying gaps and challenges in program operations.

As part of evaluation efforts, TB programs should also analyze each new TB case and each TB-related death to determine whether the case or death might have been prevented. On the basis of such assessments, the program can develop and implement new policies to reduce the number of preventable cases and deaths and prepare annual reports based on these assessments in collaboration with community-based organizations and professional societies. These reports should document the extent and nature of the TB incidence and treatment completion in the area, assess the adequacy of prevention and care measures in demonstrating program effectiveness, and provide recommendations for program improvements. A TB program might determine that an outside review by experts from the state health department, CDC, local lung associations, or other TB experts could be helpful in determining methods for improving program performance and community TB control and for providing support for major changes (e.g., significant restructuring or acquisition of new resources).

### Quality Improvement for All TB Programs

All TB programs should collect, analyze, and use data to help guide decisions about the quality of TB services and patient outcomes. A quality improvement (QI) approach focuses on systematically assessing and improving processes and outcomes for patient care, with the overall goal of ensuring positive outcomes for patients. QI uses data to assess the strengths and weaknesses in delivering care to patients and measuring clinical outcomes.

Key components of a QI program include

- conducting assessments that identify areas requiring improvement based on established standards;
- collecting and analyzing data to establish measurable baseline indicators;
- developing a plan with specific interventions for addressing identified problems and gaps;
- educating staff regarding problems and proposed interventions, with input from relevant staff;
- implementing interventions at the patient level by ensuring that care is patient centered at the program level and by ensuring consistency with standards of practice, evidence-based guidelines, and established agency policies;
- reassessing the identified concerns by using measurable indicators;
- using data to measure the effectiveness of the interventions;
- disseminating findings to relevant staff, administration, and other stakeholders; and
- modifying methods used to assess and measure the effectiveness of the interventions.

Assessment and implementation methods will vary across programs. Improving the quality of patient care, ensuring positive outcomes for patients, and increasing efficiency of the care delivery system are common goals. To be effective, QI should be an ongoing process that involves managers, staff, and patients.

### External TB Program Evaluation

The primary purpose of conducting an evaluation is to influence decisions. By using results of the evaluation, decision-makers might decide to continue, change, expand, or end a program or associated activity. Evaluation design is dynamic, changing as programs develop and real-world challenges emerge. Therefore, the first task for the evaluator or evaluation team is to define the intended audience and develop measurable evaluation questions. The intended audience will influence the type of evaluation questions to be addressed. For example, a local health department official might be interested in the number of persons successfully treated for LTBI in an outpatient clinic setting, whereas an official responsible for program funding is probably more interested in cost and availability of anti-TB medications.

The objective of the evaluation team is to use the best approach available that yields the most accurate results with available resources for conducting the evaluation. The evaluator or evaluation team should then be able to communicate results accurately and clearly to diverse audiences who might lack a complete understanding of program complexities and their potential impacts. A step-by-step guide to program evaluation is available from CDC’s TB website (*32*).

TB program staff apply analytic methods in different ways to assess program performance. Potential designs are available that can be used to answer the evaluation questions, and no single correct method exists. A systematic review of literature or contact with a CDC program consultant can identify additional information and tools useful for successfully answering evaluation questions of interest.

### TB Program Evaluation Network: TB Programs in States and Large Cities 

Program staff should share lessons learned with other programs and consider publishing results for the larger public health community. One forum available to all TB programs is the TB Program Evaluation Network (TB PEN). TB PEN comprises state and local TB program evaluation personnel designated as focal points (i.e., staff in each jurisdiction directly funded by CDC who are responsible for TB program evaluation activities) and other TB program staff who are working with or interested in program evaluation. TB PEN focal points participate in bimonthly conference calls to share program evaluation successes and challenges to assist one another in improving the quality of their TB program activities. The TB Education and Training Network (TB ETN) is a networking initiative that facilitates communication and collaboration among TB professionals interested in TB education and training and enhances the capacity of training and education staff in TB control programs. TB PEN collaborates with TB ETN on a biennial conference that enables TB PEN focal points to learn from in-person educational sessions, scientific research lectures, poster presentations, and program evaluation updates at local, state, tribal, and federal levels and from networking with program evaluation colleagues. The TB PEN steering committee also maintains a website that contains program evaluation and quality improvement resources (*33*).

## Laboratory and Other Testing

Numerous advances in TB prevention and control have occurred within laboratory science since this report was last published in 1995. Two types of immunologic-based test methods are available for detecting *M. tuberculosis* infection in the United States: the Mantoux TST and IGRAs. The addition of IGRA as a new test for TB infection has been groundbreaking. Unlike traditional TSTs, IGRAs differentiate between infection with *M. tuberculosis* versus the effect of bacillus Calmette-Guérin (BCG) vaccine. TB epidemiology in the United States identifies the majority of new TB disease cases among persons from countries with high TB incidence with uniform BCG vaccination requirements. IGRAs allow identification of TB infection regardless of BCG status. However, the TST is still a valid test for TB infection (Supplementary Appendix E; https://stacks.cdc.gov/view/cdc/90289).

New molecular diagnostic tests identify both *M. tuberculosis* and genetic mutations that identify some degree of potential drug resistance within hours of specimen receipt, without waiting for culture-based DSTs. Use of whole-genome sequencing can identify *M. tuberculosis* and genetic mutations associated with drug resistance. These modalities allow for earlier identification of *M. tuberculosis* and initiation of recommended therapy for TB, thus resulting in decreased transmission of *M. tuberculosis*. However, specimens should still be cultured for phenotypic drug susceptibility.

Because available tests and methods of analysis are constantly changing, detailed descriptions and uses are provided online (Supplementary Appendices; https://stacks.cdc.gov/view/cdc/90289). TB programs should have access to the most updated tests, with results reported as they become available, for managing complex cases of disease, remove persons from isolation in a timely fashion, and identify linked cases. Laboratories should report test results, including negative results, to the public health TB program in addition to the ordering clinician. Tests used for assessing TB disease and monitoring treatment progress are explained in detail (Supplementary Appendix D; https://stacks.cdc.gov/view/cdc/90289), and their use is discussed in the following section.

## Identification, Management, and Treatment of Persons with LTBI

### Importance of Screening, Testing, and Treatment for LTBI

LTBI is the presence of *M. tuberculosis* organisms (tubercle bacilli) without signs and symptoms or radiographic or bacteriologic evidence of TB disease. Therefore, persons with LTBI do not experience clinical illness; they are asymptomatic, and their infection is not transmissible. The only evidence of infection might be a reaction to a TST or a positive IGRA test. In persons with LTBI, TB infection can persist for decades, and those with *M. tuberculosis* infection can remain at risk for progressing to TB disease, especially if the immune system becomes impaired. An estimated 13 million persons have LTBI in the United States (*34*).

Since the early 1980s, TB prevention and control in the United States has expanded with treatment of persons with LTBI to prevent TB disease. Approximately 80% of the active TB disease cases in the United States are believed to be caused by reactivation of LTBI (*34*,*35*). As TB disease rates in the United States decrease, finding and treating persons at high risk for LTBI has become a higher priority and a cornerstone strategy for TB elimination. LTBI treatment is important because it can substantially reduce the risk that persons infected with *M. tuberculosis* will progress to TB disease. However, LTBI treatment can be associated with adverse events; therefore, the goal of preventive therapy is to treat those for whom prophylaxis for LTBI carries substantially more benefit than potential harm.

### Principles for Risk Assessment, Testing, and Treatment for LTBI

TB risk assessment is an essential TB prevention and control strategy that helps detect persons with LTBI who can benefit from treatment. Because TB risk assessment de-emphasizes testing of groups who are not at high risk for TB, it can help reduce wasted resources and prevent nonessential treatment. Programs should pursue testing for LTBI only if diagnostic evaluation can be performed, a course of therapy can be prescribed, and therapy is likely to be completed.

Guidelines provide recommendations for groups who should be screened, tested, and treated for LTBI (*36*,*37*). USPSTF guidelines provide recommendations for incorporating TB prevention into routine primary care (*26*). A risk assessment based on local or state epidemiology of TB infection and disease should be prepared and made available to stakeholders and providers. Clinical, epidemiologic, and behavioral risk factors for TB disease and infection have been documented and are included in the joint guidelines from the American Thoracic Society (ATS), Infectious Diseases Society of America (IDSA), and CDC. Considering these risk factors in two categories can be helpful: 1) risk factors for TB exposure or 2) risk factors for progression to active TB disease after becoming infected (*37*).

TB programs should retest a person who previously tested negative only if new risk factors occur after the previous assessment. Risk factors might include new close contact with a patient with infectious TB or new immunosuppression but also can include foreign residence or foreign travel in certain circumstances (e.g., extended duration, likely contact with persons with infectious TB, or high prevalence of TB in a travel location). Health care providers should check with local TB control programs for recommendations regarding local epidemiology, regulations, legal mandates, and patient populations when setting TB testing policies (see Role of Health Departments and Overall Planning and Policy Components) (Supplementary Appendix F; https://stacks.cdc.gov/view/cdc/90289).

### Repeat Screening of Persons at Continued Risk for Exposure to TB Disease

The frequency of repeat testing depends on a person’s degree of risk for exposure to TB disease as determined by locally generated data. Facilities should compile and analyze their epidemiologic and programmatic data and work with local and state health departments when making those decisions.

### Test Selection and Interpretation

Selecting a test for TB infection depends on the situation and the patient (Supplementary Appendix D; https://stacks.cdc.gov/view/cdc/90289). Among patients with an initial negative test who are at risk for poor outcomes, use of both TSTs and IGRAs can increase sensitivity for detecting *M. tuberculosis* infection. Using both tests is not recommended unless the test that is initially used is negative. Persons who might be considered at risk for poor outcomes include those with HIV infection, children aged <5 years, and persons with other immunosuppressive conditions (*38*,*39*). Routine testing with either TST or IGRA among populations at low risk for TB infection is not recommended.

### Interpreting TST Results

Based on the sensitivity and specificity of the TST and the prevalence of TB in different groups, three cutoff levels have been recommended for defining a positive tuberculin reaction: ≥5 mm, ≥10 mm, and ≥15 mm of induration. The criteria for tuberculin skin positivity, by reaction induration cutoff level and risk group, are provided (Box 5).

BOX 5 Criteria for tuberculin skin positivity, by reaction induration cutoff level and risk group*

**≥5 mm of induration**

Persons with human immunodeficiency virus (HIV) infection

Close contacts of a person with infectious tuberculosis (TB)

Persons with chest radiographs consistent with previous untreated TB

Organ transplant recipients

Other immunosuppressed persons^†^

**≥10 mm of induration**

Recent immigrants

Injection drug users

Residents or employees of congregate settings

Mycobacteriology laboratory personnel

**≥15 mm of induration**

Persons with no known risk factors for TB

**Source:** CDC. Targeted tuberculin testing and treatment of latent tuberculosis infection. MMWR Recommend Rep 2000;49(No. RR-6).

* From highest (≥5 mm) to lowest (≥15 mm) risk for developing active TB disease. These cut points apply both to adults and children and can be modified on the basis of local epidemiology.

^†^ Patients taking the equivalent of >15 mg/day of prednisone for 1 month or those taking tumor necrosis factor-α antagonists or other immunomodulators.

### Interpreting IGRA Test Results

QuantiFERON test results are reported as positive, negative, or indeterminate, and T-SPOT test results are reported as positive, negative, indeterminate, or borderline. CDC recommends that the laboratory provide both quantitative and qualitative results (*39*).

### Deciding on Treatment

TB disease should be ruled out after any positive TB test, with a thorough physical examination, history, chest radiography, and when indicated, bacteriologic studies. Determining whether a patient has a previous history of treatment for LTBI or TB disease and performing a risk assessment for TB infection are vital for determining the benefits of treatment (see Principles for Risk Assessment, Testing, and Treatment for LTBI).

If a patient has a positive test result and TB disease is ruled out, the patient should be considered for LTBI treatment. If the person accepts and is able to receive treatment for LTBI, the clinician should develop a plan of treatment with the patient to ensure adherence. However, if the patient refuses or is unable to receive treatment for LTBI, the clinician should educate the patient about the signs and symptoms of TB disease and about the need for rapid medical evaluation if TB signs or symptoms occur.

### Treatment Regimens for LTBI

Since the 1995 publication of “Essential Components of a Tuberculosis Prevention and Control Program” (*1*), numerous important studies of shorter LTBI treatment regimens have been published. These studies present high quality of evidence, efficacy, effectiveness (including better treatment completion rates and fewer side effects) supporting the use of shorter regimens for LTBI (*40*–*44*). CDC and NTCA have recently published guidelines for the treatment of LTBI that favor use of shorter rifamycin-based regimens when possible (*45*). Many public health programs are using these shorter regimens (Supplementary Appendix F; https://stacks.cdc.gov/view/cdc/90289).

## Identification, Management, and Treatment of Persons with TB Disease

Effective treatment of persons requires both a tailored medical management plan and a patient-focused case management plan. After disease has been verified or is strongly suspected, both plans should be coordinated so that the most optimal care is provided to the patient, family, and community.

### Available Services for TB Control

TB control programs should ensure that the services needed for evaluating, treating, and monitoring TB patients are readily available in each community. In certain areas, these services might be provided directly by the state TB program. In other areas, local TB programs or health care professionals, with supervision and consultation from the city or state TB program, provide patients’ treatment services. The policies, procedures, and laws specified at the beginning of this report (see Overall Planning and Policy Components) provide guidance for managing care for persons with TB disease. Although patients might undergo the majority of their evaluation and treatment in settings other than the health department, the major responsibility for monitoring and ensuring the quality of all TB-related activities in the community lies with the health department as part of its duties in protecting public health.

### Protocols for TB Case Management and Treatment

The public health goals of TB patient management are to initiate treatment promptly and ensure completion of effective therapy to cure disease, reduce transmission, and prevent development of drug-resistant TB. These goals are achieved through case management. The TB program should have protocols in place for TB case management and treatment (*46*,*47*). The TB control program, in conjunction with the patient’s health care provider, is responsible for ensuring that the TB patient completes the recommended treatment for TB disease in a timely manner.

### Identifying Persons with Clinically Active TB Disease: Diagnostic Methods

TB programs should be familiar with and have access to new diagnostic tools (e.g., blood-based IGRAs, nucleic acid amplification tests [NAATs], and other diagnostic tests as they become available). The laboratory appendix (Supplementary Appendix D; https://stacks.cdc.gov/view/cdc/90289) includes information regarding specific tests. Sputum and other specimens from suspected sites should be obtained as soon as possible for acid-fast bacillus smear and culture, rapid identification of *M. tuberculosis* complex by using NAATs, and initial molecular testing within the limits of the guidelines (*48*). Each patient should receive a medical evaluation and chest radiography, with additional imaging of the affected area, if not the lungs. Additional tests, including assessment for HIV infection status, should be performed, depending on the patient’s medical history and current condition. A medical regimen should be prescribed on the basis of patient clinical and epidemiologic characteristics (*48*).

### Management and Treatment: Medical and Case Management Collaboration

#### Case Management Plan

Case management for TB disease includes patient-centered activities (e.g., DOT for medications, assessment of side effects, and patient monitoring) and other public health activities. The case management team should work closely with those providing medical management to ensure optimal care for each patient. Public health workers in TB programs play an integral role in helping patients complete TB treatment through the case management process. Case management provides patient-centered care for treatment completion and ensures that all public health activities related to stopping TB transmission are completed. This includes ensuring that each patient is educated about TB and its treatment, the importance of treatment adherence, and that contacts should be elicited and evaluated. This patient-centered approach can help ensure successful treatment and public health outcomes because it emphasizes a tailored approach that addresses both the patient’s clinical and social concerns.

#### Patient Interview

Within 3 working days after the case is reported, a health department worker should visit the patient in the hospital or at home to conduct an interview, initiate patient education, identify contacts, make referrals for medical evaluation, and detect possible problems related to adherence to therapy. An initial treatment and monitoring plan should be developed and implemented within 1 week of diagnosis. This treatment plan should be reviewed regularly and modified as needed when additional relevant information becomes available (e.g., DST results) or when the patient’s care is transferred from one provider to another.

When developing and implementing a treatment plan, TB programs should work closely with health care providers from local hospitals, drug treatment centers, HIV clinics, correctional facilities, dialysis centers, health maintenance organizations, private physicians’ offices, and other facilities where TB patients receive medical care. TB programs should fulfill their mandated responsibilities and also respect the relationship between the patient and the primary health care provider. Other resources describe case management for persons with TB disease (*46*,*47*).

#### Case Management Team

In addition to the medical and case manager, team members might include clinic supervisors, outreach workers, health educators, nurses, nurse practitioners, physician assistants, pharmacists, physicians, and social workers. The patient is always a member of the team; family members might assist as available or interested. Specific responsibilities might be assigned to other team members; however, the case manager is ultimately responsible for ensuring that needed activities are performed. The specifics of this team, including size and number of members and function of each member, vary by jurisdiction and local needs.

Although certain patients might undergo their evaluation and treatment in settings other than the health department (e.g., hospitals or correctional facilities), the health department is legally obligated to monitor and ensure the quality of all TB-related activities within a health jurisdiction. Thus, all TB patients should be assigned case managers, whether they receive TB care in health department clinics or from private providers.

#### Medical Manager

A specific clinician should be responsible for decisions regarding patient medications, testing, and assessment of progress throughout treatment. That clinician should provide medical oversight of all patient care and thus should have an excellent understanding of TB disease and its treatment, the effect of comorbidities on TB treatment, and drug-to-drug interactions (*49*).

#### Case Manager

A specific health care worker (i.e., a case manager) should be assigned primary responsibility for ensuring that all treatment and public health activities associated with the TB patient are completed. Although one person is assigned primary responsibility, case management can involve a team of persons who collaborate to provide continuity of care. The case manager is responsible for ensuring the following activities are completed for all TB patients to whom they are assigned:

- Establishing a trusting relationship with the patient;
- Educating the patient about TB and its treatment;
- Ensuring that treatment and monitoring plans are in place;
- Ensuring the patient adheres to and completes treatment;
- Identifying contacts of a patient with infectious TB and providing testing and treatment as needed for all contacts;
- Expanding the contact investigation as necessary when results from initial investigations become available; and
- Conducting quality assurance through routine systematic review of patient progress.

### Assessing and Promoting Adherence

Methods for promoting adherence to therapy should be tailored to the patient’s needs, lifestyle, social support system, and beliefs about health. An assessment of these factors should be included in developing a case management plan (*46*,*47*). TB programs should educate patients about the causes and effects of TB, dosing and possible adverse reactions of their medications, and the importance of taking their medications according to the care plan. To facilitate adherence, the plan should use short-course treatment regimens and fixed-dose combinations, if such regimens and combinations are recommended and available. A welcoming and respectful atmosphere within the clinic setting is fundamental to maintaining adherence.

The case manager should conduct an assessment of risk for nonadherence to treatment. TB programs should consider treating all patients with DOT, which is the standard of care in the United States (*50*). With DOT, a health care provider or other responsible person observes the patient swallowing each dose of anti-TB medication. DOT can be administered with daily or intermittent regimens and can be administered to patients in an office or a clinic setting or by an outreach worker in the patient’s home, place of employment, school, or other mutually agreed-upon place. In certain instances, DOT might be administered by the staff of correctional facilities or drug treatment programs, dialysis center staff, home health care personnel, staff of maternal and child health facilities, or responsible community members. New technologic methods (e.g., video DOT) might be used to promote adherence to treatment when in-person DOT is not feasible (*51*,*52*).

Incentives and enablers should be available for enhancing adherence to therapy. An incentive is an inducement or reward that serves as motivation for a desired action (e.g., a gift card for a local shop after completion of the first 2 weeks of treatment or a small toy for a child who takes the medication). An enabler is an item or action that removes barriers for achieving a desired outcome (e.g., transportation passes to get to the clinic or assistance with rent payments to prevent a person from becoming homeless).

Health care professionals, including private practitioners, who become aware of a TB patient who has demonstrated an inability or unwillingness to adhere to a prescribed treatment regimen should immediately consult the health department. The TB program can assist in evaluating the patient for the causes of nonadherence to therapy and provide assistance (e.g., outreach worker services) to enable the patient to complete the recommended therapy. If the patient still does not adhere to treatment, the health department should take action based on local and state laws and regulations. This entails issuing a health officer order for DOT or seeking court-ordered DOT or detention for patients who are unwilling or unable to complete treatment and who have infectious TB or for those who are at risk for becoming infectious or experiencing drug-resistant TB. A list of recommended legal resources for TB programs has been developed (*19*).

Additional services might be needed to facilitate continuity and completion of therapy. Social workers, interpreters, and referral sources should be available in the clinic or easily accessible to the patients. To ensure that patients receive treatment until they are cured, TB programs should make use of available legal authority and facilities available to isolate and treat patients who have infectious TB (see Overall Planning and Policy Components). When all less restrictive measures have failed, TB programs should be prepared to use any available legal authority to detain patients unwilling or unable to complete their treatment. This authority also might apply to nonadherent patients who no longer have infectious TB but whose disease might again become infectious or develop drug resistance. Procedures and plans should be established to ensure that patients in isolation or detention have safeguards for due process (e.g., how to request release from detention) and have their basic needs met (e.g., food, basic supplies, and other necessities).

### Medical Management Plan

Although the majority of medical treatment plans for TB disease begin with the standard four-drug regimen (rifampin, isoniazid, pyrazinamide, and ethambutol [RIPE]), patient-specific drug regimens should be considered on the basis of the patient’s history. For example, a history of exposure to persons with multidrug-resistant TB (MDR TB) might change the initial medication recommendations. A history of bladder cancer treatment in a patient with disseminated disease might lead the clinician to consider that the cause of the TB disease is *Mycobacterium bovis* BCG rather than *M. tuberculosis*.

Clinicians should take a thorough medical history, conduct a complete physical examination to detect TB disease outside the lungs, and assess the patient’s history of exposure to TB disease and other factors in the patient’s history to select the best initial drug regimen. Subsequent decisions about the patient’s regimen depend on the results of drug susceptibilities, side effects experienced by the patient, disease progression, and any new information about the patient’s history and exposures that are discovered after the initial evaluation.

### Initiation of the Treatment and Case Management Plans

As soon as patient specimens and bacteriology are obtained and TB disease is diagnosed or suspected, a clinician should start treatment and ensure the TB case is reported to the health department. TB programs should send smear-positive respiratory specimens for TB identification and molecular diagnostic testing to test for genetic mutations that are surrogates for drug-resistant TB (*48*). TB programs should start TB treatment either empirically or on the basis of laboratory findings such as molecular analysis or DST results. Because the majority of TB disease in the United States is pansusceptible, patients usually can be started empirically on the standard four-drug treatment regimen noted previously in the most recent version of the ATS/CDC/IDSA TB treatment guidelines (*48*).

### Clinic Services

Clinic services provided by TB programs, if available, should be accessible and acceptable to community members served by the clinic. Clinic hours should be convenient and ideally might include evening or weekend hours for persons who work or attend school. The clinic should be easily accessible by public transportation, or transportation should be provided, if possible. Intervals between the time of referral and the time of appointment and waiting times in the clinic should be kept to a minimum. In busy TB clinics or multipurpose clinics, priority should be given to persons with TB disease or being evaluated for TB disease and to persons receiving TB medications. Clinic services, including diagnostic evaluation, medications, and transportation, should be provided regardless of the patient’s ability to pay. The clinic should have staff who speak the same language and have similar cultural and socioeconomic backgrounds as the community served by the clinic, or the clinic should employ persons trained to work in cross-cultural settings. Language interpretation services should be available.

### Clinical Consultative Services

Expert medical consultation should be available for management of all TB patients, including those who have drug-resistant TB. These consultative services should be available to the TB program and health care providers in the community. The consultation might be provided by a staff member of the TB program or by a local or regional consultant collaborating with the health department. Consultative services are also provided through CDC’s TB Centers of Excellence (*53*).

### Drug-Resistant and MDR TB

MDR TB should be considered in patients with of a history of previous TB treatment or who are from a country with high MDR TB rates (*37*). Treatment initiation decisions can be guided by results of molecular testing. Molecular testing results might be obtained by sending specimens to the CDC Molecular Detection of Drug Resistance (MDDR) service (*54*) and public health laboratories. Specimens might also be sent to National Jewish Health in Denver, Colorado, or to other laboratories, for a fee. Certain instruments can test for isoniazid resistance, as recommended by WHO. Rifampin resistance can be tested by the GeneXpert or through MDDR testing.

Newer drugs (e.g., bedaquiline) have been approved for MDR TB treatment. Drugs that are approved for other bacterial infections (e.g., fluoroquinolones and linezolid) also are important drugs for treating MDR TB. Although shorter treatment regimens for MDR TB are being investigated, describing those trials is beyond the scope of this report. TB controllers should keep abreast of new developments related to drug-susceptible and drug-resistant TB. Medical consultation should be sought for any questions related to TB treatment and especially for decisions regarding MDR TB treatment regimens. Certain jurisdictions have specific MDR TB consultative services; consultation might also be obtained from the TB Centers of Excellence. A guide to testing and treatment for drug-resistant TB is available from the Curry International Tuberculosis Center’s website (*55*). On the basis of broad individual patient data meta-analyses, newer guidelines for treating drug-resistant TB have been developed by ATS, CDC, the European Respiratory Society, and IDSA (*56*).

### Referral System for Other Medical Problems

A system should be in place to facilitate referral of TB patients for evaluation and treatment of other medical problems, including those conditions that can affect the course or outcome of TB treatment (e.g., HIV infection, underlying malignancy, diabetes mellitus, and substance abuse). Consultants should see referred patients in a timely fashion, and the consultant’s assessment and recommendations should be made available promptly to the referring health care provider. If patients receive care in more than one setting, treatment should be coordinated with the other health care providers to ensure continuity and completion of therapy, minimize drug interactions, and avoid duplication of efforts. The TB program takes primary responsibility for ensuring TB treatment and monitoring for adherence. TB programs should refer patients with infectious TB with recommended respiratory precautions and notify the receiving health care provider or transport personnel that the patient has an aerosol-transmissible disease.

### TB Care in Inpatient and Other Clinical Settings

The TB program and all clinical settings should develop and implement protocols for ensuring rapid reporting of known or suspected TB cases to the health department having jurisdiction. Regardless of the patient’s ability to pay, TB programs should make accommodations available for any TB patient requiring inpatient hospital care for TB-related conditions. The facility should have effective infection control measures in place to prevent transmission of TB infection within the hospital (*18*). For example, the hospital should have provisions that allow patients with suspected or confirmed infectious TB disease to be separated from other patients. Although ideally such patients should be placed in an airborne infection isolation room, if such a room is unavailable, a room with effective general ventilation should be used, with use of air cleaning technologies (e.g., a portable high-efficiency particulate air [HEPA] filtration system). Medical staff knowledgeable about the management of TB patients should be available to assist in patient care while the patient is hospitalized. In addition, medications should be available in the facility so that the patient can start or continue therapy in the hospital. Diagnostic services (e.g., radiology and mycobacteriology) should be available for monitoring the patient for the response to treatment. The patient should also be monitored for adverse events and for other existing or new medical conditions.

**Inpatient Care.** Staff at inpatient settings might be unfamiliar with standards of TB treatment (e.g., DOT for all medications). Ingestion might not be documented, or the doses might not be counted in the overall dose count for treatment; therefore, the TB program and the patient are best served by the inpatient staff performing DOT and documenting actual ingestion of the medications. As soon as possible after admission, a representative from the TB program should visit the patient in the hospital to identify contacts, collect information for the initial treatment plan, and ensure that no obstacles to the patient’s follow-up care exist.

Discharge planning from the hospital begins as soon as the patient is admitted. TB programs should work with the hospital to facilitate TB patient discharge. Some jurisdictions allow the discharge of patients with infectious TB into the community, provided that DOT has been initiated and that the patient is tolerating medications, is being discharged to a setting that facilitates continued care and treatment while minimizing potential exposure of others, and has an appointment for follow-up. A contract between the patient and the health department to maintain noninstitutional home isolation might be required. A hospitalized patient should be reported to the health department; in many jurisdictions, approval from the health department must be obtained before discharge (*57*–*59*).

**Other Settings: Coordinating Care with Other Health Care Providers and Facilities.** TB patients often receive care in multiple settings, including HIV clinics, drug treatment centers, correctional facilities, hospitals, nursing homes, or primary care clinics. When patients move among these different settings, continuity and completion of treatment can be compromised, unless a system for coordinating care exists. To provide and coordinate continuous TB treatment and to facilitate transfers of care, TB programs should communicate regularly with providers and facilities involved in TB patient care, including hospitals, infection control practitioners, private practitioners, community clinics, correctional facilities, homeless shelters, and drug treatment centers.

## Epidemiologic Investigation

### Contact Investigation

Contact investigation is a key component of any TB program, and the purpose is to actively identify additional persons who might have TB disease or LTBI. The process involves interviewing persons with TB disease to identify anyone with whom they have close, prolonged contact. This might include persons sharing the same household, close friends, or close work contacts. After these persons are identified, they undergo an evaluation that might include LTBI testing, ruling out TB disease, and identifying persons with a history of previous TB disease or infection. Contacts are tested according to CDC and NTCA guidelines (*60*). Contacts who have a positive test (e.g., TST or IGRA) or have suspected or confirmed TB disease are offered treatment for LTBI or TB disease as indicated. Many of these persons receive case management until therapy completion. Contact investigations encompass all aspects of TB control, including surveillance, case identification, case management, infection control, and prevention. Protocols should be in place to assist TB program staff in performing contact investigations, including determination and documentation of the infectious period to establish priorities for the investigation.

Contact investigations should be conducted by using a concentric circle model, with an initial focus on those at highest risk. Persons at highest risk include those with prolonged exposure to an infectious person, those at risk for progression to active TB disease, or those at high risk for transmitting disease to others. The priority, speed, and extent of a contact investigation should be guided by the likelihood of transmission on the basis of the characteristics of the source patient, environment, and exposed persons. This activity can identify undiagnosed active TB cases, in addition to persons with LTBI.

TB program staff should begin a contact investigation as soon as they are notified of a suspected or confirmed case of infectious TB. A contact investigation is initiated for suspected or confirmed cases of respiratory TB (i.e., pulmonary, laryngeal, or pleural, when indicated).

To identify persons accurately as contacts, program staff should determine the infectious period for the index patient. An infectious period is calculated to identify the period during which the exposure is most intense (on the basis of patient clinical characteristics) and when it stops (on the basis of treatment initiation). TB programs should calculate an 8- to 10-week window period. Preventive treatment of presumed TB infection is recommended during the window period for contacts who are at increased risk for progression to TB disease (i.e., children aged <5 years and immunocompromised persons). Decisions to treat persons beyond the window period might be made for certain persons. TB programs should communicate with providers who are not in the health department. Contacts might choose to be tested by their own provider rather than by TB program staff. In that instance, communicating with providers to ensure exposed persons are evaluated in a timely manner and retested after the window period, if indicated, is crucial.

TB programs should assess transmission after evaluation and testing of the closest contacts, with expansion of a contact investigation, if indicated. If expansion is indicated, the next circle of contacts might be in congregate settings such as workplaces, schools, correctional facilities, hospital or health care settings, and shelters or other settings providing services for homeless persons. These investigations can be complicated and can include educational sessions for exposed and unexposed persons, communication with senior-level health department staff, and possible interest by media outlets. Ultimately, ensuring that exposed persons are evaluated for TB infection and disease is part of the health department’s mandated responsibility for protecting the public’s health.

### Genotyping and Clustering

TB genotyping is a laboratory-based approach used for analyzing the genetic material of *M. tuberculosis* that distinguishes different *M. tuberculosis* strains (*61*,*62*). When combined with epidemiologic data, genotyping can help identify persons with TB disease involved in the same chain of recent transmission. TB genotyping helps distinguish between persons whose TB disease is the result of TB infection that was acquired in the past and those with recently or newly acquired infection with development of TB disease. TB genotyping can be helpful during a conventional contact investigation. Use of genotyping can discover unsuspected transmission associations between TB patients by identifying unknown transmission settings or interjurisdictional transmission. Genotyping can also establish criteria for outbreak-related case definitions, identify additional persons with TB disease involved in an outbreak, determine completeness of contact investigations, distinguish recent reinfection from relapse, and detect laboratory cross-contamination.

When two or more *M. tuberculosis* isolates are matched by genotyping methods, they are referred to as a genotype cluster. Members of the same genotype cluster are assumed to have the same strain, which can be a surrogate for recent transmission and the need for further epidemiologic investigation. Genotyping information, epidemiologic linkages, and DST results can help distinguish recent transmission from reactivation of TB infection. Analysis of clustered cases can assist in prioritization of resources for further epidemiologic investigation. Clusters can become outbreaks if transmission is ongoing in the community. Members of the same genotype cluster are assumed to have the same strain, which might indicate recent transmission and the need for further epidemiologic investigation. Investigation of patient demographic, geographic, and clinical data; genotyping; and DST results can help determine epidemiologic links between patients.

### TB Outbreaks

A TB outbreak investigation is initiated when transmission that is higher than expected is detected given the population demographics, the local epidemiology in a health jurisdiction, or the strain genotype. An outbreak investigation typically requires public health resources beyond those that are routinely needed for TB control. TB outbreaks are defined on the basis of local epidemiology of the jurisdiction and are relative to the local context. A recommended definition consistent with either of the set of the following criteria can be used: 1) two or more contacts are identified as having active TB disease, or 2) two or more cases that have occurred within 1 year of one another are discovered to be linked.

An outbreak investigation is conducted urgently and places greater demands on the health department. To the extent possible, investigations should be guided by surveillance data that include all available clinical, epidemiologic, and genotyping information. The decision to initiate an investigation should be discussed with senior-level staff to ensure that the necessary resources will be available.

### Investigations of Laboratory Cross-Contamination

Public health programs have an important role in identifying and investigating laboratory cross-contamination, which can also be referred to as a false-positive investigation. A false-positive *M. tuberculosis* specimen is a positive culture that is not the result of disease but is caused by contamination of a clinical device, a clerical error, or laboratory cross-contamination during processing. Identification of cross-contamination provides an opportunity to correct equipment or processes that are responsible for the false-positive culture, correct an erroneous medical diagnosis, and discontinue unnecessary TB treatment and contact or source case investigations. Identification of cross-contamination also allows for removal of data related to a patient’s incorrect diagnosis from local and national surveillance systems. False-positive cultures might be identified through review of patients with only a single positive culture reported, review of genotyping results, or clinician suspicion.

### Source Case Investigations

Source case investigations focus on identifying the source of infection or disease for young children (i.e., aged ≤5 years). TB disease in a U.S.-born child who has never lived or traveled outside the United States is a sentinel event. The assumption is that any child with TB must have been exposed recently to someone close to the child and that the person likely has unrecognized TB disease. Young children with TB usually do not transmit TB to others, and their contacts are less likely to be infected because of exposure to them (*56*). Seeking a source case follows the same overall procedures as a standard contact investigation. Parents or guardians, referred to as associates, usually are the best informants. Attention should be focused on all associates who have symptoms of TB disease. A source case investigation should begin with the closest associates (e.g., household members).

Source case investigations should also be considered for children aged ≤5 years with one or more risk factors for LTBI, most often birth or extended stay in a high-prevalence country, who test positive for LTBI. These investigations allow TB programs to identify high-risk households where persons might have undiagnosed or untreated LTBI or TB disease. Although the yield for finding TB disease in these investigations is usually low, family members might have LTBI and be candidates for treatment. Each jurisdiction should determine its policy for conducting source case investigations on the basis of available resources.

## Training and Education

TB programs should provide education and training in the clinical and public health aspects of TB for all TB program staff. Resources are available to assist TB programs with fulfilling training and education responsibilities (Supplementary Appendix G; https://stacks.cdc.gov/view/cdc/90289).

### Education and Training for All TB Program Staff

TB program staff should receive education at regular intervals regarding their particular responsibilities in the program and should demonstrate proficiency in those areas. Training needs should be tailored to the type of TB staff receiving training (e.g., clinical staff, public health nurses, TB program managers, communicable disease investigators, community health workers, or health educators). The following aspects should be included in staff education and training:

- **TB knowledge and education.** TB programs should provide tailored training and education for all TB program staff at the time of employment, at regular intervals during their employment, and as needed to ensure that staff maintain an accurate, updated level of knowledge about TB infection and disease, diagnosis and treatment, public health practice, management and evaluation skills, laboratory test algorithms and methods, and other related topics. Many training and education products have been developed and made available through CDC (Supplementary Appendix G; https://stacks.cdc.gov/view/cdc/90289). Staff should receive ongoing education on the basis of their particular responsibilities and be knowledgeable about other staff responsibilities (e.g., use of interpreters). All staff should also receive cultural-sensitivity training. The Patients’ Charter for Tuberculosis Care (*63*) describes patients’ rights and responsibilities and should be made available to staff and to patients in languages spoken by the patient population, if available. Local and state epidemiology of TB and emergence of new populations or settings of high risk also might necessitate additional training or retraining of staff.
- **Periodic needs assessment.** TB programs should undertake staff training needs assessments. These assessments should include a determination of the practitioners’ knowledge, skills, and attitudes regarding any planned TB control interventions.
- **Contact investigation training.** TB programs should provide staff training regarding how to conduct a contact interview and how to conduct a contact investigation. The interrelated tasks in a contact investigation require personnel in the health department to fulfill multiple functions and skills. The education needs for all aspects of the investigation process, including medical abstraction, patient interviewing, cultural competency, maintaining patient confidentiality, and how to perform TSTs, should be assessed continuously.
- **TB surveillance.** TB programs should ensure that relevant staff understand the methods for performing optimal TB surveillance, including active and passive surveillance and validation.
- **Patient confidentiality.** Confidentiality is essential in several of the core components of a TB control program. Health care personnel should be educated about confidentiality concerns that are relevant during encounters between patients and health care personnel and for collecting, managing, and sharing data related to TB patients.
- **Cohort review.** TB program staff should understand the methods and content and have the skills needed to perform cohort reviews.
- **Evaluation.** TB programs should ensure that staff understand the principles of evaluation, the evaluation process, and methods needed.

### Comprehensive and Tailored TB Information for Public and Private Audiences

TB programs should provide comprehensive and tailored information about TB to public and private audiences, including health care providers in private and community settings, other staff at community health centers and hospitals, academic institutions, medical professional organizations, community-based organizations, correctional facilities, and civil surgeons. Educational activities should be tailored to these intended audiences. 

**Health Care Provider Education.** Education and communication should be prepared for health care providers to improve their knowledge and awareness of TB and to guide access to competent clinical services. Health care provider education should include

- local TB reporting laws, including reporting procedures for those suspected of having TB;
- current recommendations for TB diagnosis, treatment, and management;
- vital elements that should be applied to prevent and control TB in different settings and among populations at risk; and
- communication linkages with public health for specialized TB clinical support and resources for shared TB case management, as needed.

**Community-Based Education.** Community leaders and health policymakers, including public health officials, advocates, and other community members, should be well-informed about the need to communicate and address concerns as they arise. Important components of this education include

- local TB epidemiology, including drug resistance and populations at high risk;
- the basics of TB diagnosis and treatment;
- the need for partnering with the local public health TB sector to facilitate access to services for target populations;
- the need for advocating to support TB patients and the public health sector; and
- the need for coordinating with the public health sector to develop educational materials tailored to local populations.

### Patient and Family Education

Education of patients and their families by clinicians, TB program staff, and trusted community members is widely believed to promote acceptance and adherence to recommended TB prevention and treatment activities and legal implications; however, a defined need exists for more quality trials evaluating educational interventions with patients and their families, both for active TB and LTBI (*64*). Such education can influence patients’ decision-making regarding whether to accept and complete treatment (*65*). TB programs should enlist community-based organizations and other key informants, as needed, to determine the health beliefs, norms, and values of communities at high risk in their jurisdictions. An interpreter should be used when the patient and health care provider do not speak the same language. Education efforts should include print and electronic materials that are nonstigmatizing and sensitive to the patient’s culture, language, age, and reading level. Educational materials should be written in plain language, be understandable and visually appealing, and be provided in the patient’s language, if available. These materials should include information about

- TB and expected outcomes of treatment;
- timeline for course of treatment, sputum samples, release from isolation, and return to work;
- infectiousness and infection control;
- drugs, drug management, potential drug toxicity, common side effects, and drug resistance;
- contact investigation;
- DOT;
- potential legal implications;
- other aspects of patient care, as needed; and
- relevant information reinforcement.

## Partnerships and Collaboration

TB elimination cannot be accomplished by public health efforts alone. Because WHO estimates that approximately 25% of the world has LTBI (*66*), considerable participation from traditional and nontraditional partners is required.

### Identifying Partners and Establishing Relationships

Traditional partners include clinicians, community health centers, and lung associations. Nontraditional partners include cultural associations, religious organizations, minority health groups, public service organizations, and others. Review and analysis of TB program data will assist in identifying potential partners. Relationships should be established between the TB control program and partners of interest (*18*), and each relationship might be initiated by the partner or the TB control program.

### Defining Goals

Partners should collaborate in establishing mutual goals. Defining mutual goals is an essential element in the partnership for TB prevention and control and requires group-specific goals.

### Implementing and Evaluating Goals

After these goals are established, strategies for implementation can be developed. Any implementation strategy should include methods for evaluating each intervention’s success or failure. Developing partnerships, identifying and supporting mutual goals, and creating and implementing a plan for TB prevention and control require time, effort, and resources. Theories and practices are available for guiding these efforts, and health departments often have education programs that can offer assistance. All partnerships and collaborations are specific to the program. Although partnerships and collaboration can be challenging to build and maintain, they are essential for TB elimination in the United States (*18*).

## Research

Despite a sustained decrease in TB disease incidence in the United States from the early 1950s through 2020, with the existing tools, TB will not be eliminated by 2050 or before, as thought possible by WHO for low-incidence countries (*4*). Therefore, increasing efforts and resources is essential for combating and eliminating TB in the United States and globally. Research, including at the local, national, and international levels, is needed for answering questions that can lead to TB elimination worldwide.

Clinical and basic research are essential for providing improvements in TB diagnosis and treatment and for evaluating the accuracy and effectiveness of tests and treatments. Programs should take every opportunity to participate in local, national, and international research based on local capacity. Local and state statistics can help researchers identify questions suitable for study at the local, state, or tribal level (see Program Evaluation and Quality Improvement). For information regarding CDC TB studies, see CDC’s TB website (*67*); in addition, a summary of research activities and needs is described (Supplementary Appendix H; https://stacks.cdc.gov/view/cdc/90289).

## Conclusion

State and local health departments have the primary responsibility for preventing and controlling TB. This report provides an introduction for TB controllers regarding the essential components of a public health TB prevention, control, and elimination program and is intended as a reference tool to assist in the broad spectrum of activities that are essential for all U.S. jurisdictions to progress toward and eventually achieve TB elimination. ACET and NTCA have prepared this report to provide and update national standards for use by and assessment of individual TB control programs by TB control program managers, policymakers, and other persons evaluating TB programs.  This report also can be used to assist local programs in obtaining and maintaining adequate resources for TB control activities.  TB control program managers should continue in their collective efforts to incorporate each of the components described in this report into their program activities.
